# Human enteroviral infection impairs autophagy in clonal INS(832/13) cells and human pancreatic islet cells

**DOI:** 10.1007/s00125-020-05219-z

**Published:** 2020-07-16

**Authors:** Anya Wernersson, Luis Sarmiento, Elaine Cowan, Malin Fex, Corrado M. Cilio

**Affiliations:** 1 Unit of Molecular Metabolism, Department of Clinical Sciences, Lund University Diabetes Centre, Clinical Research Center 91:10, Jan Waldenströmsgata 35, SE-21428 Malmö, Sweden; 2 Immunovirology Unit, Department of Clinical Sciences, Lund University Diabetes Centre, Malmö, Sweden

**Keywords:** Autophagy, Beta cells, Enterovirus, Insulin secretion, Lysosomes, Type 1 diabetes, Viral replication, Virus spread

## Abstract

**Aim/hypothesis:**

Human enteroviral infections are suggested to be associated with type 1 diabetes*.* However, the mechanism by which enteroviruses can trigger disease remains unknown. The present study aims to investigate the impact of enterovirus on autophagy, a cellular process that regulates beta cell homeostasis, using the clonal beta cell line INS(832/13) and human islet cells as in vitro models.

**Methods:**

INS(832/13) cells and human islet cells were infected with a strain of echovirus 16 (E16), originally isolated from the stool of a child who developed type 1 diabetes-associated autoantibodies. Virus production and release was determined by 50% cell culture infectious dose (CCID_50_) assay and FACS analysis. The occurrence of autophagy, autophagosomes, lysosomes and autolysosomes was detected by western blot, baculoviral-mediated expression of microtubule-associated protein light chain 3 (LC3)II-GFP and LysoTracker Red, and quantified by Cellomics ArrayScan. Autophagy was also monitored with a Cyto-ID detection kit. Nutrient deprivation (low glucose [2.8 mmol/l]), amino acid starvation (Earle’s Balanced Salt Solution [EBSS]) and autophagy-modifying agents (rapamycin and chloroquine) were used in control experiments. Insulin secretion and the expression of autophagy-related (*Atg*) genes and genes involved in autophagosome–lysosome fusion were determined.

**Results:**

E16-infected INS(832/13) cells displayed an accumulation of autophagosomes, compared with non-treated (NT) cells (grown in complete RPMI1640 containing 11.1 mmol/l glucose) (32.1 ± 1.7 vs 21.0 ± 1.2 μm^2^/cell; *p =* 0.05). This was accompanied by increased LC3II ratio both in E16-infected cells grown in low glucose (LG) (2.8 mmol/l) (0.42 ± 0.03 vs 0.11 ± 0.04 (arbitrary units [a.u.]); *p* < 0.0001) and grown in media containing 11.1 mmol/l glucose (0.37 ± 0.016 vs 0.05 ± 0.02 (a.u.); *p* < 0.0001). Additionally, p62 accumulated in cells after E16 infection when grown in LG (1.23 ± 0.31 vs 0.36 ± 0.12 (a.u.); *p* = 0.012) and grown in media containing 11.1 mmol/l glucose (1.79 ± 0.39 vs 0.66 ± 0.15 (a.u.); *p* = 0.0078). mRNA levels of genes involved in autophagosome formation and autophagosome–lysosome fusion remained unchanged in E16-infected cells, except *Atg7*, which was significantly increased when autophagy was induced by E16 infection, in combination with LG (1.48 ± 0.08-fold; *p* = 0.02) and at 11.1 mmol/l glucose (1.26 ± 0.2-fold; *p* = 0.001), compared with NT controls. Moreover, autophagosomes accumulated in E16-infected cells to the same extent as when cells were treated with the lysosomal inhibitor, chloroquine, clearly indicating that autophagosome turnover was blocked. Upon infection, there was an increased viral titre in the cell culture supernatant and a marked reduction in glucose-stimulated insulin secretion (112.9 ± 24.4 vs 209.8 ± 24.4 ng [mg protein]^–1^ h^–1^; *p* = 0.006), compared with uninfected controls, but cellular viability remained unaffected. Importantly, and in agreement with the observations for INS(832/13) cells, E16 infection impaired autophagic flux in primary human islet cells (46.5 ± 1.6 vs 34.4 ± 2.1 μm^2^/cell; *p* = 0.01).

**Conclusions/interpretation:**

Enteroviruses disrupt beta cell autophagy by impairing the later stages of the autophagic pathway, without influencing expression of key genes involved in core autophagy machinery. This results in increased viral replication, non-lytic viral spread and accumulation of autophagic structures, all of which may contribute to beta cell demise and type 1 diabetes.

**Figure:**
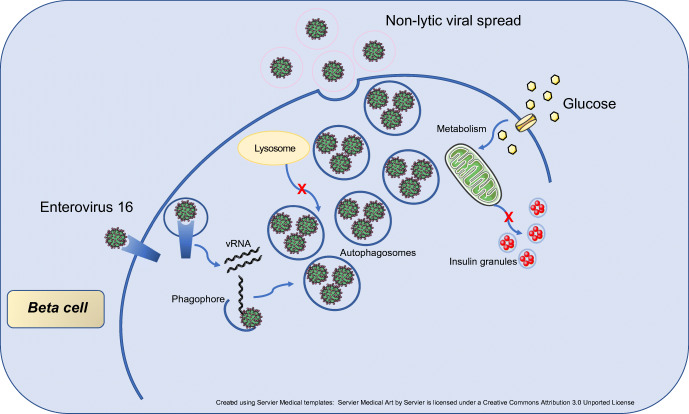
Graphical abstract

**Electronic supplementary material:**

The online version of this article (10.1007/s00125-020-05219-z) contains peer-reviewed but unedited supplementary material, which is available to authorised users.

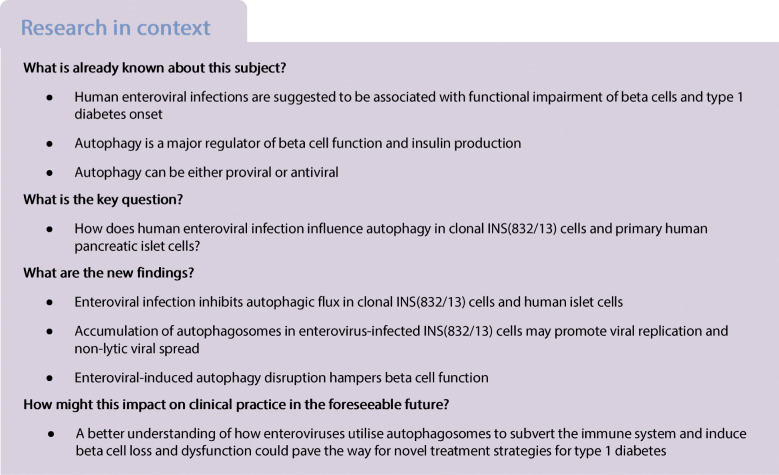



## Introduction

Type 1 diabetes is one of the most common chronic diseases occurring in childhood and adolescence, thought to be triggered by viral infections. Evidence supports a scenario where an enteroviral infection could spread to the pancreas and establish a persistent infection within beta cells [1–3]. Such infections could trigger autoimmunity and type 1 diabetes development in genetically susceptible individuals [1]. How enteroviruses can establish a persistent infection in the pancreas is unclear as the underlying mechanisms of enterovirus-mediated beta cell dysfunction remain unknown.

Macro-autophagy (hereafter referred to as autophagy) is a process that ensures cellular survival under stressful conditions related to metabolic stress, nutrient deprivation and viral infections [4]. In addition, autophagy regulates insulin homeostasis and plays a critical role in resolving beta cell stress [5].

Autophagy begins with the formation of a double-membrane structure, the phagophore, that engulfs cytosolic constituents into vesicles (autophagosome). Lipidation of cytosolic microtubule-associated protein light chain 3 (LC3)I produces a membrane-associated form (LC3II), essential for autophagosome formation. The autophagosome formation process is tightly regulated by more than 32 autophagy-related (*Atg*) genes [6], and autophagosomes subsequently fuse with lysosomes to generate autolysosomes with a single membrane morphology, where the sequestered cargo, including LC3II and the scaffolding protein sequestosome 1 (SQSTM1)/p62 (referred to as p62), are degraded by proteases. Autophagosome–lysosome fusion can be achieved through soluble *N*-ethylmaleimide-sensitive factor attachment protein receptors (SNAREs) and anchoring proteins (i.e. lysosome-associated membrane protein 2 [LAMP2], syntaxin 17 [STX17] and UV radiation resistance-associated protein [UVRAG]) [7]. The overall dynamic process is termed autophagic flux [8].

Enteroviruses are obligate intracellular pathogens and have evolved strategies to escape lysosomal degradation [9, 10]. In addition, enteroviruses have co-opted the autophagy machinery as a proviral host factor favouring viral replication [11]. Infections of poliovirus, Coxsackievirus and enterovirus 71 in RD-A (human rhabdomyosarcoma) and HeLa cells, induced double-membrane vesicles resembling autophagosomes, which promoted viral RNA replication [12–14]. Similar findings were observed in vivo, where autophagosome-like vesicles acted as membrane scaffolds for viral replication in cardiomyocytes and pancreatic acinar cells of mice infected with coxsackievirus B3 [15, 16].

Even though both in vitro and in vivo studies suggest that enteroviruses are capable of subverting the autophagic machinery to facilitate their own replication, the role of beta cell autophagy, enteroviral infection and its impact on the pathogenesis of type 1 diabetes still remains unexplored. In fact, determining the molecular basis of enteroviral infection in beta cells may aid in understanding the pathogenesis of type 1 diabetes. As such, targeting autophagy or viral infections may provide novel strategies to prevent or treat the disease. Thus, the aim of this study was to investigate the impact of enteroviral infection on autophagy in clonal INS(832/13) cells and primary human pancreatic islet cells.

## Methods

### Cells and virus

Mycoplasma tested INS(832/13) [17] were cultured at 37°C and 5% CO_2_ in complete RPMI1640 (11.1 mmol/l glucose supplemented with 2 mmol/l l-glutamine, 1 mmol/l sodium pyruvate, 50 μmol/l β-mercaptoethanol, 10 mmol/l HEPES and 10% FBS; Sigma Aldrich, St Louis, MO, USA).

Human islets from 11 non-diabetic individuals (six male and five female donors, BMI 31.03 ± 4 kg/m^2^, HbA_1c_ 40.3 ± 3.9 mmol/mol or 5.3 ± 0.4%; see human islets checklist in the electronic supplementary material [ESM]) were used in evaluation of autophagy, viral replication and viability analysis. Islets were hand-picked under a stereo microscope and dissociated in Ca^2+^-free medium (20 min at 37°C), by pipetting, into single cells. Cells were cultured at 37°C and 5% CO_2_ in RPMI1640 medium with 5.5 mmol glucose and 10% FBS (vol./vol.) (Sigma Aldrich) for 24 h.

Echovirus 16 (E16) was isolated from the stool of an individual who developed type 1 diabetes autoantibodies [18]. Virus identity was confirmed with type-specific antisera and VP1 sequence primer pairs 187 (VP1; 5′-ACIGCIGYIGARACIGGNCA-3′) and 011 (2A; 5′-GCICCIGAYTGITGICCRAA-3′) (Thermo Fisher Scientific, Waltham, MA, USA). E16 stocks were prepared by infecting 90% confluent green monkey kidney (GMK) cells until cytopathic effects were observed. Centrifugation removed debris (400 *g* for 10 min) and titres were determined by end-point dilutions in microwell cultures of GMK cells, expressed as a 50% cell culture infectious dose (CCID_50_)/ml according to the Spearman–Karber method [19]. UV-irradiation was used to inactivate the virus, with a 15 W UV lamp at 10 cm distance for 45–60 min. Inactivation was verified by titration in GMK cells.

Human islets were acquired from the Human Tissue Laboratory in Malmö, Sweden via the Nordic Network for Clinical Islet Transplantation, Uppsala, Sweden. The study was approved by the ethics committees in Malmö and Uppsala, Sweden.

### Viral replication

INS(832/13) cells were seeded at 1 × 10^5^/ml in 24-well plates and infected the next day with E16 at the indicated multiplicity of infection (MOI). Plates corresponding to specific time points were infected and incubated. Following adsorption for 2 h at 36°C, one plate was taken out and cells were washed twice with PBS removing unattached virus, to determine viral background levels. For remaining plates, 1 ml of fresh RPMI1640 medium with 2% FBS/well was added. Cells and supernatant were harvested at 24, 48 and 72 h post infection (hpi). Supernatant samples were used to determine extracellular infection, after centrifugation. Adherent cells were rinsed twice with PBS and frozen (−80°C). Intracellular infection was assessed from cell pellets after three freeze–thaw cycles to release the virus. Viral particle dose (CCID_50_) was determined both in supernatants and cell pellet by end-point dilutions in microwell cultures of GMK cells [19]. To confirm intracellular viral replication, cells were harvested by mechanical scraping. Detached cells were stained with double-stranded RNA (dsRNA)-specific mAb J2 (SCICON, English and Scientific Consulting, Szirak, Hungary) and data were acquired using a CytoFlex Flow Cytometer (Beckman Coulter, Brea, CA, USA). Results were analysed with CytExpert 2.0 Software (Beckman Coulter).

Dispersed human islets were cultured (50,000 cells/well) in non-attach 24-well plates and infected with E16 at the indicated MOI. Infectious medium was left on cells to minimise loss due to low cell adhesion. Supernatant samples were harvested at 0 h (directly after infection) and thereafter at an interval of 24 h for 3 days. The CCID_50_ of each sample was determined by end-point titration in GMK cells [19].

### Starvation and drug treatments

For glucose starvation, INS(832/13) and islet cells were grown for 24 h in complete RPMI1640 medium containing 2.8 mmol/l glucose (low glucose, LG). Controls/non-treated (NT) INS(832/13) cells were grown in complete RPMI1640 medium containing 11.1 mmol/l glucose. Cells were also incubated with 0.5 μmol/l rapamycin, dissolved in 0.04% DMSO (an autophagy inducer; Enzo, Plymouth Meeting, PA, USA [24 h incubation]), 10 μmol/l chloroquine (a lysosomal inhibitor; Enzo [24 h incubation]) or in amino-acid- and serum-free buffer (Earle’s Balanced Salt Solution [EBSS], Sigma Aldrich [4 h incubation]).

### Viability

3-(4,5-Dimethylthiazol-2-yl)-2,5-diphenyltetrazolium bromide (MTT) assay (Thermo Fisher) was used to determine cell viability of INS(832/13) cells. Quantification of apoptosis was performed in plated cells (8-well chambers; Nalgene Nunc, Thermo Fisher). Briefly, cells were washed with PBS and incubated with annexin V, Alexa Fluor 488 conjugate (Life Technologies, Stockholm, Sweden) for 5 min at room temperature in the dark. Cells were washed twice in PBS and then fixed for 10 min in 2% paraformaldehyde, washed twice again in PBS and mounted with VECTASHIELD containing DAPI (VectaLabs, Murarrie, QLD, Australia). Thereafter cells were visualised and counted using an epi-fluorescence microscope (Olympus, BX60, Tokyo, Japan), with a digital camera (Nikon DS-2Mv, Tokyo, Japan).

Cell membrane integrity was assessed by lactate dehydrogenase (LDH) cytotoxicity assay kit (Thermo Fisher) according to the manufacturer’s guidelines. Islet cell viability was assessed using 7-aminoactinomycin D (7-AAD; Sigma Aldrich). Islets were dissociated using accutase (BD Bioscience, East Rutherford, NJ, USA) at 37°C for 5 min. RPMI 1640 cell culture medium (FBS 10%) was added to stop the process. Viability was determined using a CytoFlex Flow Cytometer (Beckman Coulter) and data analysed with CytExpert 2.0 Software (Beckman Coulter). Cells were first gated for singlets using forward scatter height (FSC-H) by FSC area (FSC-A) followed SSC-A by FSC-A to exclude false positive events. Following this, cells were further analysed for their uptake of 7-AAD to determine live versus dead cells. Each analysis included fluorescence minus controls to ensure correct gating.

### Western blot analysis

INS(832/13) cells were lysed in RIPA buffer (50 mmol/l Tris-HCl, pH 7.4, 150 mmol/l NaCl, 1% NP40, 0.5% sodium deoxycholate, 0.1% SDS supplemented with complete, EDTA-free Protease Inhibitor Cocktail [Roche, Mannheim, Germany]). 10 μl of lysed cells was used for protein analysis (bicinchoninic acid [BCA] kit, Pierce Biotechnology, Rockford, IL, USA). Proteins (15–25 μg) were loaded onto 12% Criterion XT Bis-Tris Protein gels and blotted (0.2 μm PVDF membranes BioRad, CA, USA). Membranes were incubated (1 h at room temperature) in blocking solution (5% skimmed milk in Tris-buffered saline pH 7.5 containing 20 mmol/l Tris-HCl, 150 mmol/l NaCl, with 0.1% Tween 20, Sigma Aldrich), followed by overnight incubation (4°C) with primary antibodies anti-LC3I/II (#4108, 1:1000) anti-p62 antibody (#5114, 1:1000), anti-autophagy-related (ATG)7 (#8558, 1:1000) (Cell Signaling), anti-STX17 (Sigma HPA001204, 1:500) and anti-LAMP2 (Abcam ab203224, 1:500). α-Tubulin or β-actin was used as loading control (anti-α-tubulin, T5168, 1:1000, Sigma Aldrich; anti-β-actin, Cell Signaling 3700S, 1:1000). Blots were incubated (1 h) with secondary horseradish peroxidase (HRP)-linked goat anti-rabbit IgG (SC2004, 1:10,000, Santa Cruz). Immunoreactivity was detected by chemiluminescence. Quantification was performed using relative densities, normalised to α/β-tubulin bands from the same gel (Biorad, Hercules, CA, USA), and shown as arbitrary units (a.u.).

### LC3II and LysoTracker detection

INS(832/13) and islet cells were seeded in 8-well chambers (Nalgene Nunc, Thermo Fisher). After 48 h, cells were either left untreated (NT cells) or infected with E16. Cells treated with LG were included as a positive control for autophagy. Thereafter, LC3II-GFP (1:400) (Premo Autophagy sensors BacMam 2.0, Life Technologies, OR, USA) was introduced for 18 h. Two hours prior to the end of the incubation period (i.e. at 16 h), LysoTracker Red DND-99 (1:2000) (Life Technologies) was added. LysoTracker Red stains lysosomes and autolysosomes. Islet cells were counterstained with polyclonal guinea pig anti-insulin (1:500) (DAKO, Jena, Germany) and secondary antibody Alexa Flour 594 anti-guinea pig IgG (H+L) conjugate (Thermo Fisher). Cells were washed twice in PBS, pH 7.4, fixed with 4% paraformaldehyde, washed twice again in PBS and mounted in VECTASHIELD Mounting Medium with DAPI (nuclear staining).

### Autophagy analysis

Image data were acquired with an ArrayScan XTI Live High Content Platform, with a ×20 magnification (Cellomics, Thermo Fisher). For image analysis, 300 validated cells for each treatment group were analysed with Thermo Scientific Co-localisation BioApplication, to obtain the LC3II, LysoTracker and co-localisation fluorescence area per cell (in μm^2^). Using an epi-fluorescence microscope we acquired representative images (Olympus, BX60, Tokyo, Japan), captured with a digital camera (Nikon DS-2Mv, Tokyo, Japan).

Flow cytometric detection of autophagosomes in cells was performed using a Cyto-ID Autophagy Detection Kit (Enzo Life Science, New York, NY, USA). After treatments, cells were collected by centrifugation and resuspended in 1 × assay buffer. CYTO-ID Green stain solution was added to each sample, then incubated for 30 min at 37°C in dark. After washing the cells with 1 × assay buffer, data were acquired using a CytoFlex Flow Cytometer (Beckman Coulter) and analysed with CytExpert 2.0 Software (Beckman Coulter). Cells were first gated for viable cells (FSC-A vs SSC-A). Cells were then gated to exclude apoptotic cells (FSC-A vs FSC-H). Using Cyto-ID fluorescence in the FITC-A channel, autophagic vesicles were quantified and plotted as cell counts in superimposed histograms.

### qPCR of INS(832/13) cells

Total RNA was extracted from cells using RNeasy mini kit (Qiagen, Venlo, the Netherlands). cDNA was obtained by reverse transcription with Maxima First strand cDNA synthesis kit for RT-quantitative (q)PCR (Thermo Fisher). mRNA levels were quantified using a Maxima Probe/ROX qPCR Master Mix (Thermo Fisher) on an ABI PRISM 7900 (Applied Biosystems ViiA Real-Time PCR System, Life Technologies, Foster City, CA, USA). Samples were run in triplicate for each assayed gene, and presented as the fold change in gene expression normalised to the endogenous reference genes (*Ppia*, *Polr2a* and *Hprt*; Applied Biosystems, Sweden) and relative to the control condition (2^−ΔΔCt^ method).

### Insulin secretion assay

Glucose-stimulated insulin secretion (GSIS) was performed in 24-well plates where INS(832/13) cells were infected 24 h prior to assessment. Cells were washed and pre-incubated for 2 h in secretion assay buffer (SAB), containing (in mmol/l): 2.8 glucose, 114 NaCl, 4.7 KCl, 1.2 KH_2_PO_4_, 1.16 MgSO_4_, 25.5 NaHCO_3_, 20 HEPES, 2.5 CaCl_2_ and 0.2% BSA. Afterwards, cells were incubated in SAB with either low (2.8 mmol/l) or high (16.7 mmol/l) glucose for 1 h; each condition was run in triplicate. Aliquots from each well/condition were used to measure insulin (Rat Insulin ELISA, Mercodia, Uppsala, Sweden). Total protein content was extracted and measured using the BCA assay kit (Pierce Biotechnology).

### Statistical analysis

Statistical analyses were performed using Prism software (with GraphPad Prism version 7). Experiments were performed a minimum of three times, unless otherwise stated. All Cellomics ArrayScan quantifications and apoptosis measurements were analysed using ordinary one-way ANOVA, with correction for multiple comparison (Tukey’s). Gene expression, viral titres and viability assays were analysed using unpaired Student’s *t* test with Welch’s correction. Insulin secretion was analysed with non-parametrical Mann–Whitney *U* test. Data are presented as means ± SEM and a *p* value of ˂0.05 was considered significant in all experiments (*p ˂ 0.05, **p ˂ 0.01, ***p ˂ 0.001). All experiments were performed and analysed in a randomised and blinded fashion when possible. Outliers were identified using Grubbs test for outliers.

## Results

### E16 infects INS(832/13) cells and human islet cells without influencing viability

**Fig. 1:**
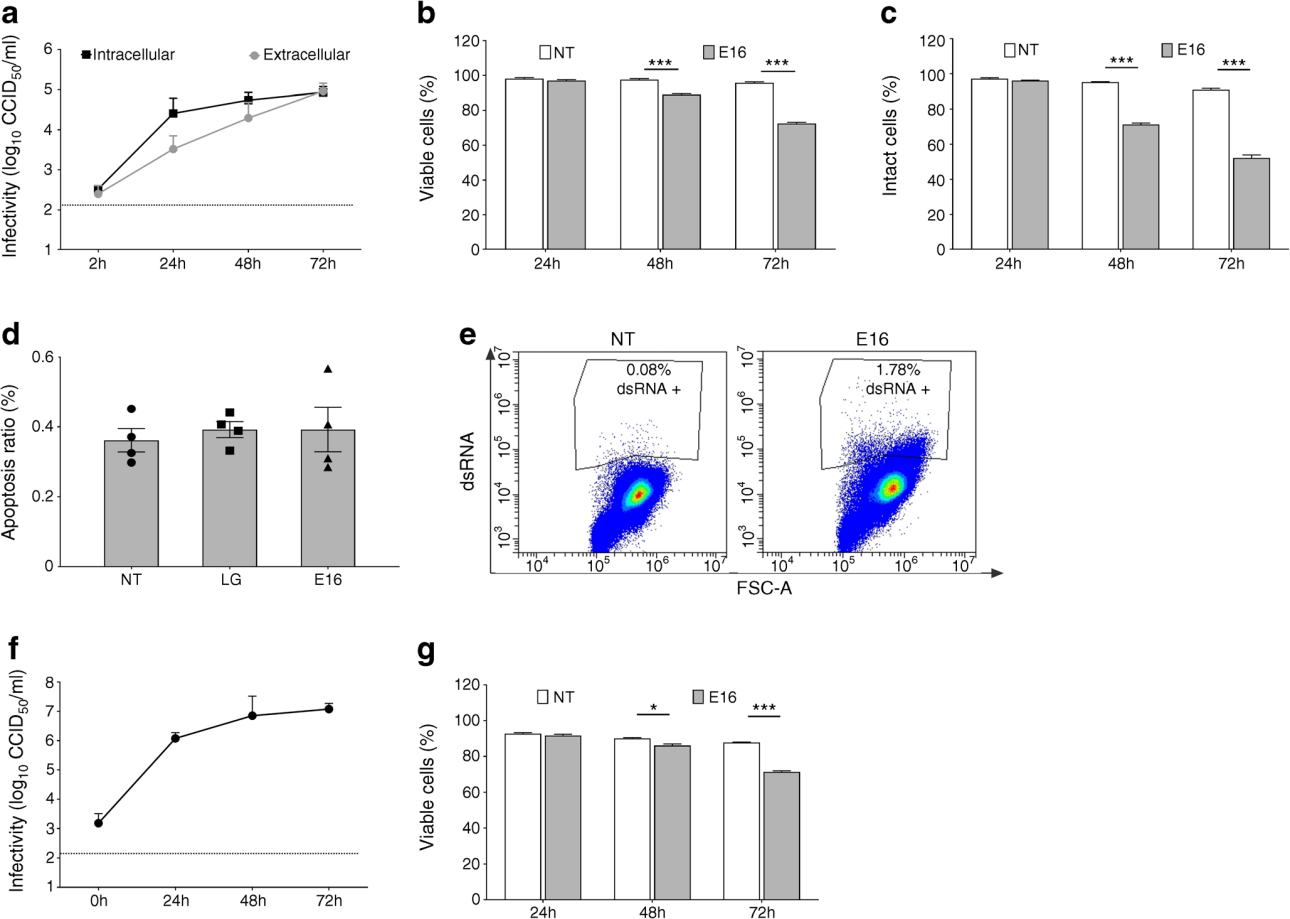
Infectivity and viability in INS(832/13) cells and human islet cells infected with E16. Increase over time of intracellular virus production and extracellular virus release determined by CCID_50_ in cell pellets and corresponding cell culture supernatant following infection of cells with E16 (MOI 0.1) (**a**). Cell viability at indicated time points after E16 infection (MOI 0.1) compared with NT cells (**b**). Plasma membrane integrity of cells (LDH leakage) at indicated time points after E16 infection (MOI 0.1) compared with NT cells (**c**). Ratio of apoptotic cells quantified by annexin V staining in NT, E16-infected or LG-treated cells after 24 h (**d**). Flow cytometry analysis of intracellular double-stranded (ds) RNA in NT-treated and E16-infected cells at 24 hpi (**e**). Extracellular virus release from islets cells at indicated time points after E16 infection (MOI 0.1) (**f**). Viability of dissociated islet cells at indicated time points after E16 infection (MOI 0.1) compared with NT cells (**g**). Results include data from 3–4 independent experiments, with each measurement performed in triplicate. Data are presented as mean ± SEM; *p < 0.05, ***p < 0.001

We determined intracellular and extracellular virus release over time in E16-infected INS(832/13) cells (MOI 1, 0.1, 0.01). In parallel, we monitored viability and plasma membrane integrity. This revealed an increase in viral titres above background levels at all MOIs tested (Fig. 1a and ESM Fig. 1a,b). Peak titres of intracellular viral production (1.87 log_10_ CCID_50_/ml) and infectious extracellular virus (1.11 log_10_ CCID_50_/ml) showed no signs of cell death (MTT assay) or leakage of LDH 24 hpi (MOI of 0.1) (Fig. 1b,c and ESM Fig. 1c–f). In addition, E16-infected cells did not show signs of apoptosis (assessed by annexin V staining) 24 hpi (MOI 0.1), when compared with cells grown in LG medium (2.8 mmol/l) or NT cells grown in medium containing 11.1 mmol/l glucose (Fig. 1d). dsRNA, a viral intermediate during its replication cycle, was detected in E16-infected cells (Fig. 1e). This shows that, regardless of viral replication, INS(832/13) cells remained viable 24 hpi.

Next, cell viability and replication of E16 in dissociated islet cells (MOI 0.1) was assessed. Similar to E16-infected INS(832/13) cells, islets cells showed an increased viral titre in the culture medium at 24 hpi (Fig. 1f), but cell viability remained unaffected (92.7 ± 0.58 vs 91.7 ± 0.68; *p* = 0.32) (Fig. 1e). In subsequent experiments, we therefore used an MOI of 0.1 when studying autophagy in INS(832/13) cells and islets cells after 24 h.

### E16 infection hampers autophagic flux in INS(832/13) cells

**Fig. 2:**
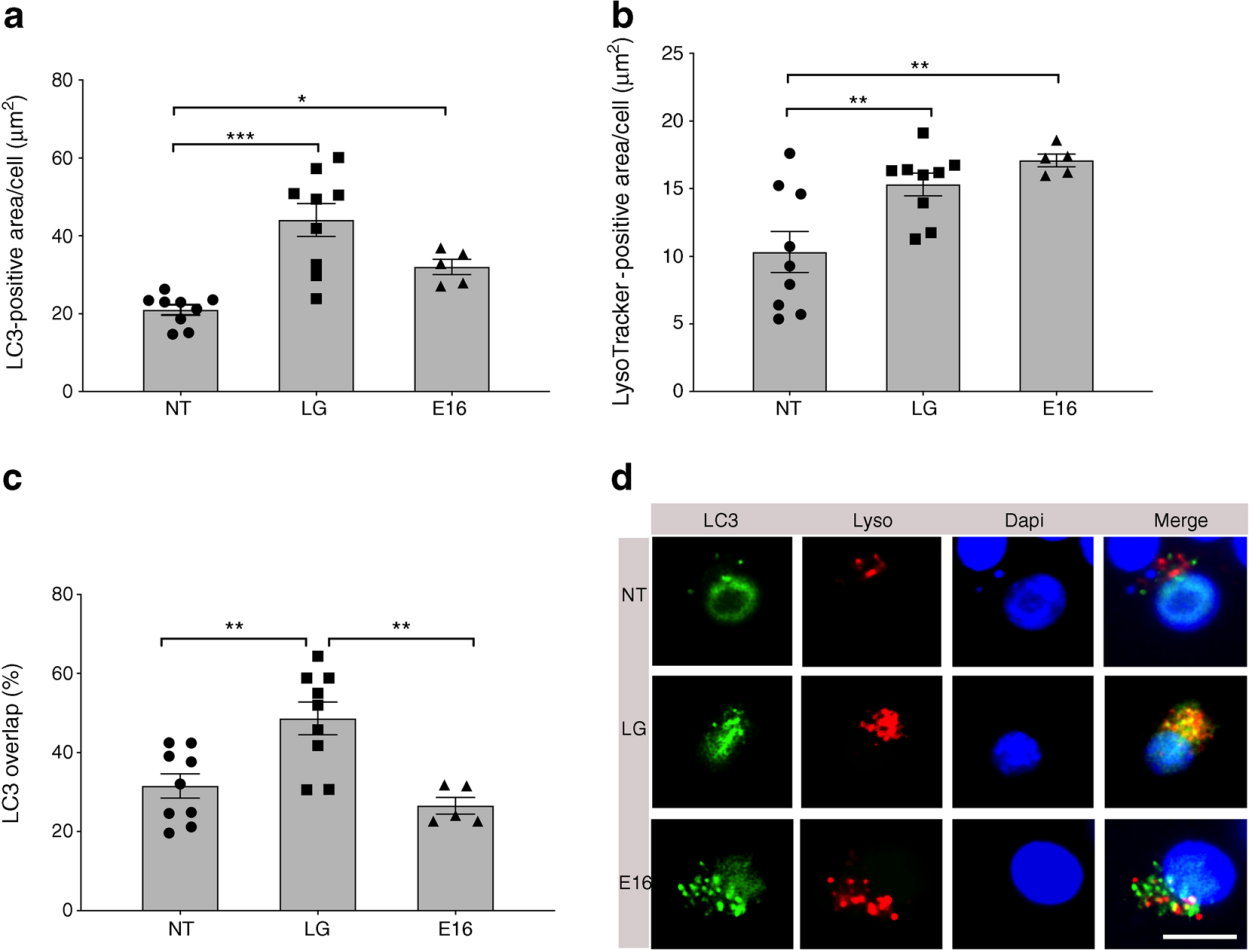
Autophagosome and lysosome detection in E16-infected INS(832/13) cells. Quantification of LC3II-GFP-positive area per cell (μm^2^) in NT- and LG-treated and E16-infected cells (**a**). Quantification of LysoTracker-positive area per cell (μm^2^) in NT- and LG-treated and E16-infected cells (**b**). Percentage of LC3II-GFP-positive areas overlapping LysoTracker-positive areas in NT- and LG-treated and E16-infected cells (**c**). LG, *n* = 9; NT, *n* = 9; and E16 *n* = 5. Representative images showing immunofluorescence staining of LC3II, LysoTracker (Lyso) and DAPI (**d**). Data are presented as mean ± SEM. *p < 0.05, **p < 0.01, ***p < 0.001

We next utilised high resolution tools to examine autophagy and flux in E16-infected INS(832/13) cells using baculoviral vectors expressing LC3II-GFP and LysoTracker Red (a lysosomal dye) [20]. As beta cells are highly dependent on ample glucose to function properly we added an LG control to these experiments as low glucose may be sufficient to induce autophagy in beta cells [21, 22]. Quantification of LC3II-GFP puncta per cell (μm^2^/cell) revealed a significant increase of autophagosomes in cells grown in LG media (44.0 ± 3.7 vs 21.0 ± 1.2; *p* = 0.0001) and E16-infected cells (32.1 ± 1.8 vs 21.0 ± 1.2; *p =* 0.05) compared with NT cells (Fig. 2a). LysoTracker Red-stained areas (in μm^2^/cell) were increased in LG-treated cells (15.3 ± 0.8 vs 10.3 ± 1.3; *p* = 0.001) as well as in cells infected with E16 (17.1 ± 0.4 vs 10.3 ± 1.3; *p* = 0.001; Fig. 2b)*.*

The percentage of LC3II-GFP-positive structures overlapping with LysoTracker-positive structures was significantly elevated in LG-treated cells compared with NT cells (48.6 ± 3.7 vs 31.5 ± 2.7; *p* = 0.004; Fig. 2c). LC3II-GFP-positive puncta overlapping LysoTracker-positive areas in E16 infected cells was similar to that of NT controls (Fig. 2c). Representative images of LC3II-GFP and LysoTracker staining support the quantitative data (Fig. 2d). Together, this suggests that autophagosomes in cells grown in LG fuse with lysosomes for subsequent degradation, while E16-infected cells display reduced fusion events leading to accumulation of autophagosomes.

### LCII ratio and p62 in E16-infected INS(832/13) cells

**Fig. 3:**
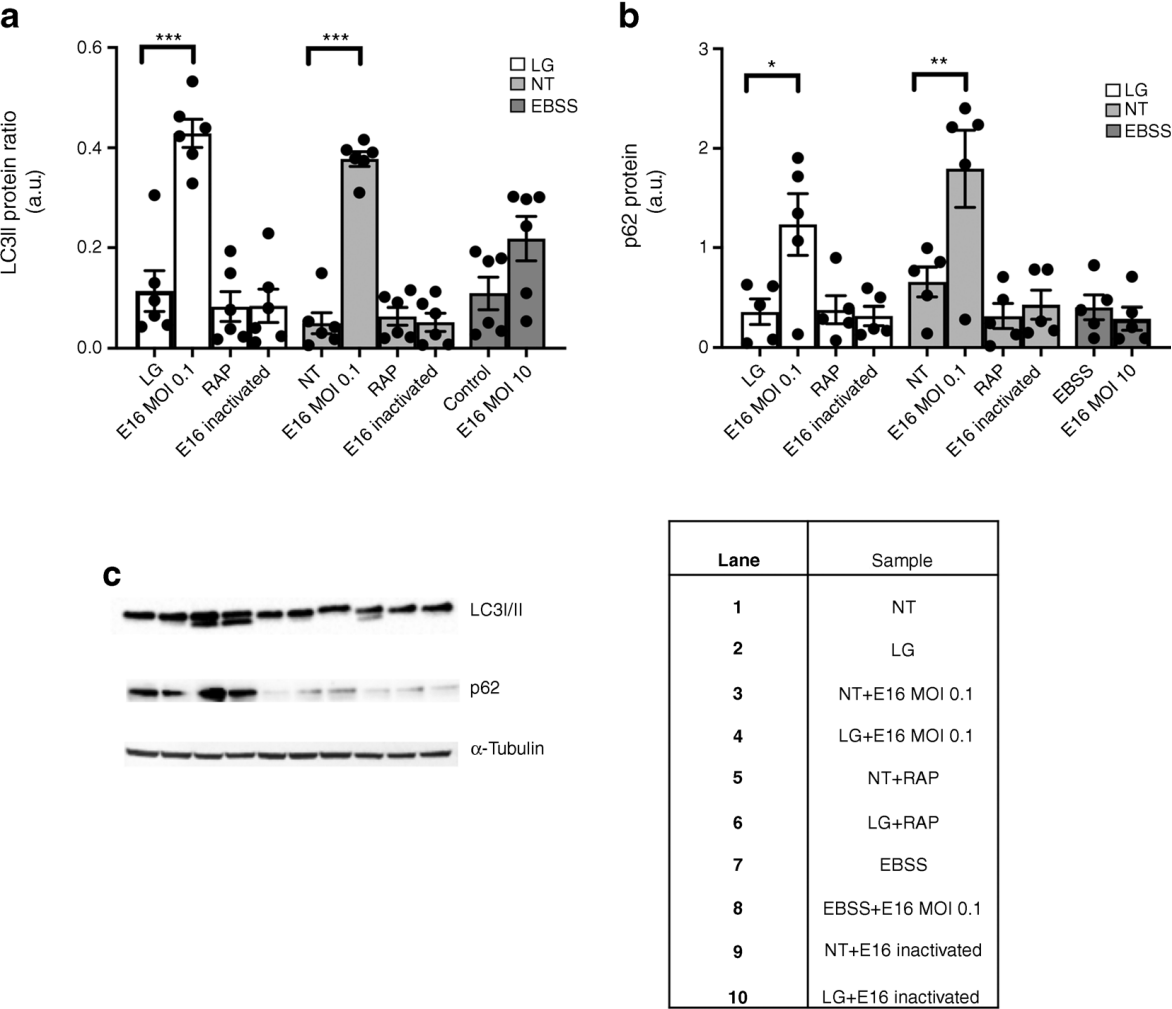
Western blot analysis of LC3II ratio and p62. Quantitative analysis of protein levels of LC3II ratio (*n* = 6) (**a**). Quantitative analysis of protein levels of p62 (*n* = 5) (**b**). Representative blots of LC3I (upper band), LC3II (lower band), p62 and loading control α-tubulin, with table indicating lanes (**c**). Data are presented as mean ± SEM of protein level relative to loading control, expressed as a.u. *p < 0.05, **p < 0.01, ***p < 0.001

To further monitor autophagy in E16-infected cells we investigated the LC3II/LC3I ratio and p62 protein levels (shown as a.u.). Cells were cultured in either LG, NT or in EBSS (amino-acid-free medium) in the presence or absence of E16 (MOI 0.1 or 10 (10 MOI for EBSS only) or UV-inactivated E16. Rapamycin-treated cells were included as a control for total autophagy flux (e.g. complete lysosomal degradation of LC3II and p62) [6, 20, 23]. We observed no changes in LC3II ratio (LC3II/[LC3I + LC3II]) in cells grown in LG and NT or with rapamycin. However, the LC3II ratios in E16-infected cells grown in LG (0.42 ± 0.03 vs 0.11 ± 0.04 *p* < 0.0001) and NT (0.37 ± 0.016 vs 0.05 ± 0.02; *p* < 0.0001) were significantly increased, compared with their respective controls (LG and NT) (Fig. 3a). Similar results were obtained for p62, where levels were increased for E16 infection in LG compared with LG control treatment. Moreover, p62 levels were also increased for E16 infection in NT compared with NT control (1.23 ± 0.31 vs 0.36 ± 0.12, *p* = 0.012 and 1.79 ± 0.39 vs 0.66 ± 0.15, *p* = 0.0078; Fig. 3b). This shows that LG and rapamycin equally well induce complete turnover of autophagy and that E16 infection effectively inhibits breakdown of p62 and LC3II, thus disrupting autophagy flux.

### E16 infection and *Atg* gene expression in INS(832/13) cells

**Fig. 4:**
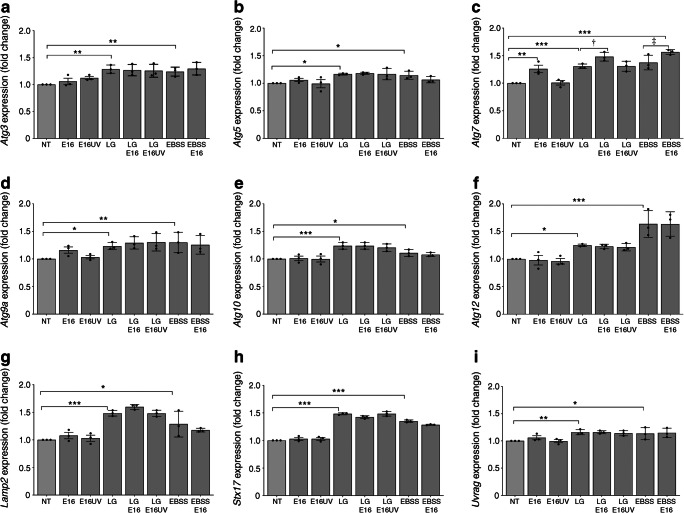
Gene expression in INS(832/13) cells. mRNA expression in cells cultured in LG, NT or EBSS in the presence or absence of E16 or UV-inactivated E16 (E16UV): *Atg3* (**a**), *Atg5* (**b**), *Atg7* (**c**), *Atg9a* (**d**), *Atg10* (**e**), *Atg12* (**f**), *Lamp2* (**g**), *Stx17* (**h**) and *Uvrag* (**i**). Data are presented as mean ± SEM (*n* = 3). *p < 0.05, **p < 0.01, ***p < 0.001

mRNA expression of *Atg* genes and genes involved in autophagosomal/lysosomal fusion was assessed in cells cultured at the same conditions as for western blot experiments (shown in Fig. 3 and discussed in the previous paragraph). mRNA expression levels of most of these genes were increased in cells either by LG or EBSS treatment, compared with NT cells. Fold change of *Atg3* (1.28 ± 0.07, *p* = 0.001 for LG treatment and 1.24 ± 0.08, *p* = 0.005 for EBSS treatment; Fig. 4a), *Atg5* (1.17 ± 0.02, *p* = 0.01 and 1.15 ± 0.07, *p* = 0.02, respectively; Fig. 4b), *Atg7* (1.30 ± 0.04, *p* = 0.0003 and 1.38 ± 0.13, *p* = 0.0001, respectively; Fig. 4c), *Atg9a* (1.23 ± 0.06, *p* = 0.03 and 1.30 ± 0.19, *p* = 0.008, respectively; Fig. 4d), *Atg10* (1.24 ± 0.06, *p* = 0.0002 and 1.11 ± 0.06, *p* = 0.04, respectively; Fig. 4e), *Atg12* (1.25 ± 0.02, *p* = 0.04 and 1.64 ± 0.24, *p* = 0.0001, respectively; Fig. 4f) *Lamp2* (1.48 ± 0.05, *p* = 0.0001 and 1.28 ± 0.23, *p* = 0.003, respectively; Fig. 4g), *Stx17* (1.48 ± 0.01, *p* = 0.0001 and 1.35 ± 0.02, *p* = 0.0001, respectively; Fig. 4h) and *Uvrag* (1.16 ± 0.04, *p* = 0.006 and 1.13 ± 0.11, *p* = 0.02, respectively; Fig. 4i). E16 infection did not alter the expression of most genes. Notably, the expression of *Atg7* was significantly increased by E16 infection at the LG condition (1.48 ± 0.08-fold, *p* = 0.02; Fig. 4c) and in cells grown in NT (1.26 ± 0.2-fold, *p* = 0.001; Fig. 4c) compared with uninfected cells. Thus, E16 stimulates the accumulation of autophagosomes by inhibiting the autophagy flux without influencing the transcription of most *Atg* genes (except for *Atg7*). It is possible, that E16 regulates these events at the protein level; therefore we examined the presence of ATG7, LAMP2 and STX17 at the protein level, but western blot analysis did not reveal any changes of these proteins in E16 infected cells (data not shown).

### Autophagosome accumulation in E16-infected INS(832/13) cells enhances viral replication and production but impairs insulin secretion

Chloroquine and rapamycin are agents that can be utilised to study specific events in the autophagic process. Chloroquine inhibits autophagic degradation in lysosomes [24] and rapamycin targets the major negative regulator of autophagy, mammalian target of rapamycin (mTOR), thus inducing autophagy [7].

**Fig. 5:**
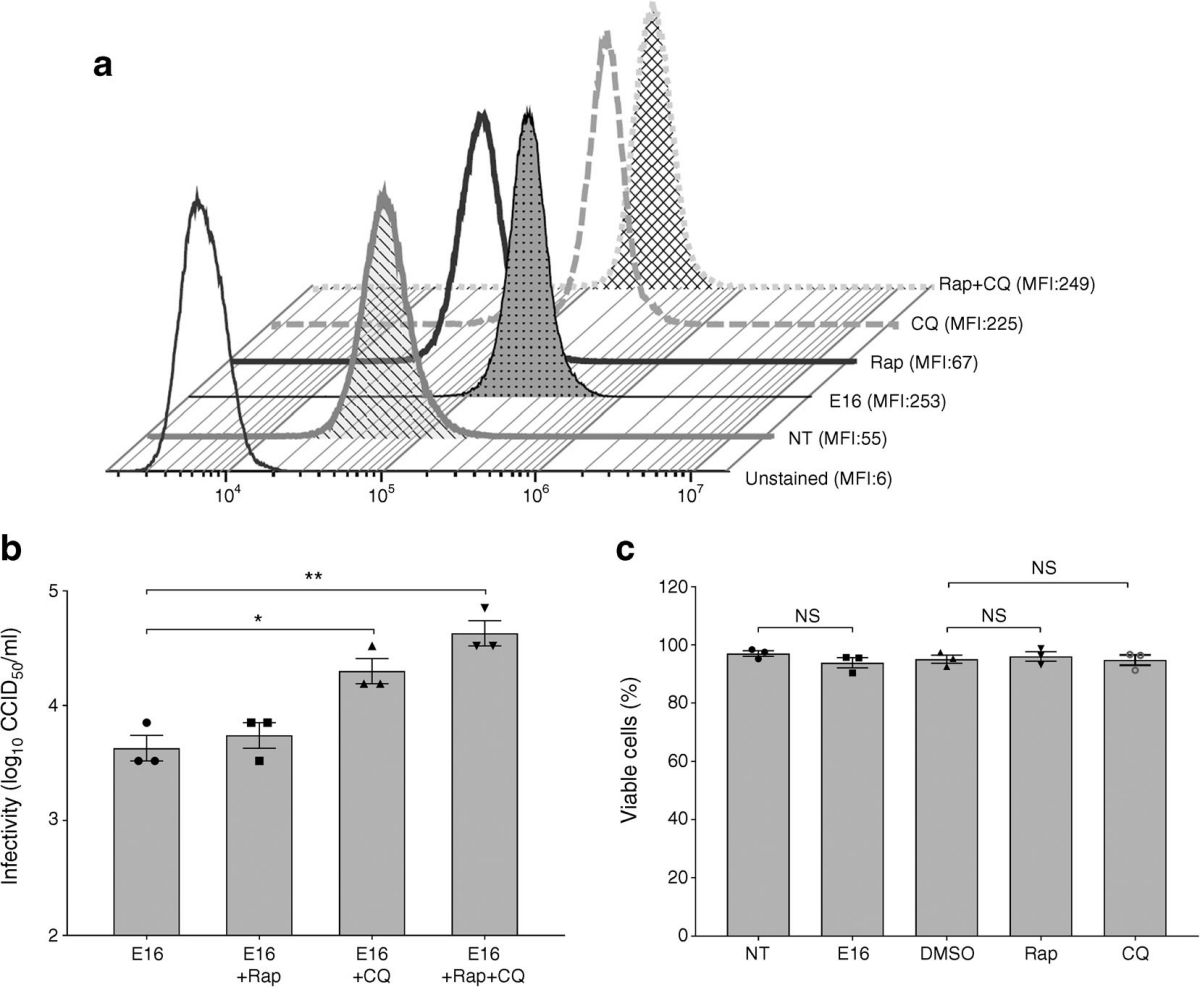
Autophagy and extracellular E16 production in E16-infected INS(832/13) cells. Cells were either left NT or treated with 0.5 μmol rapamycin (Rap), 10 μmol chloroquine (CQ) or both for 18 h. Cells were also infected with E16 (at MOI = 0.1). DMSO- (0.04%) treated cells were added as a vehicle control. Flow cytometry-based profiling of Cyto-ID Autophagy Detection Kit in infected, NT and treated cells are presented as a histogram overlay showing mean fluorescence intensity (MFI) (**a**). Cells were pre-incubated with Rap and CQ for 4 h and then infected with E16 at 0.1 MOI. After 24 hpi, extracellular virus titre in culture supernatant of cells treated with Rap, CQ or both (**b**). Cell viability of E16-infected INS(832/13) cells and INS(832/13) cells treated with Rap or CQ for 24 h (**c**). Data are representative of three independent experiments, with each measurement performed in triplicate (mean ± SEM) *p < 0.05, **p < 0.01

Cells treated with chloroquine resulted in a stronger increase in Cyto-ID fluorescence signal (mean fluorescence intensity) (Cyto-ID specifically labels autophagosomes), compared with rapamycin-treated cells (225 ± 14.4 vs 67 ± 9.5; *p* = 0.0001). Remarkably, E16 infection enhanced the Cyto-ID fluorescence signal to a similar extent as chloroquine treated cells (253 ± 18.3 vs 225 ± 14.4; *p* = 0.10) (Fig. 5a).

Pre-treatment with rapamycin and subsequent infection with E16 resulted in a slight increase in extracellular virus production, albeit not significant (3.1 ± 0.1 vs 2.8 ± 0.1; *p* = 0.75)*.* Moreover, inhibition of autophagic flux by chloroquine treatment significantly increased viral titres in the supernatant (3.6 ± 0.09 vs 2.8 ± 0.1; *p* = 0.02), compared with E16 treatment alone. Of note, combinations of chloroquine and rapamycin with E16 infection substantially increased the viral titres in the culture supernatant compared with E16 (3.9 ± 0.1 vs 2.8 ± 0.1; *p* = 0.002) and E16+rapamycin treatment alone (3.9 ± 0.1 vs 3.1 ± 0.1; *p* = 0.006; Fig. 5b). Neither viral infection nor drug treatment influenced cell viability for up to 24 h (Fig. 5c). These observations suggest that accumulation of autophagosomes promotes E16 replication and non-lytic viral release.

**Fig. 6:**
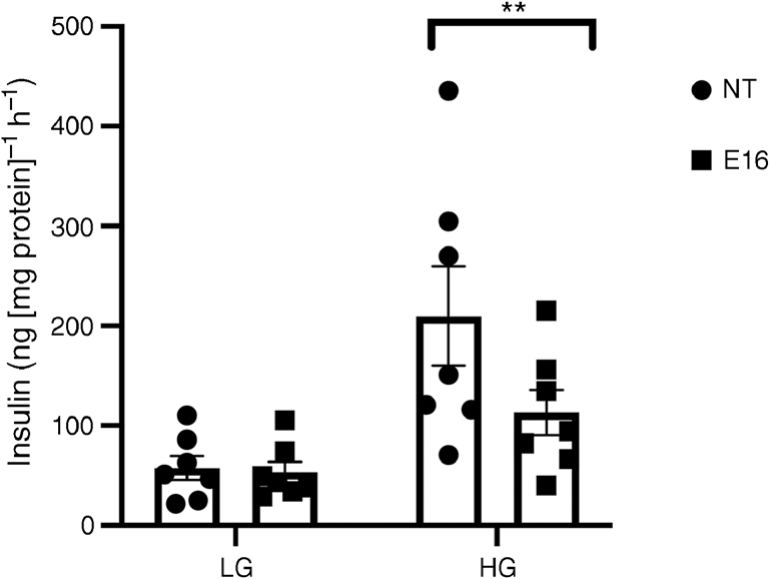
GSIS in E16-infected INS(832/13) cells. Insulin secretion in response to glucose stimulation was assessed 24 hpi by ELISA in NT (*n* = 7) and E16-infected cells (E16, *n* = 7); basal (LG; 2.8 mmol/l glucose) and glucose-stimulated (HG; 16.7 mmol/l glucose) insulin secretion are shown. Data are presented as mean ± SEM. **p < 0.01

To examine beta cell function in E16-infected cells we performed 1 h batch incubations with LG (2.8 mmol/l) and high glucose (16.7 mmol/l). Insulin secretion in E16-infected cells after stimulation with high glucose was reduced (112.9 ± 22.6 vs 209.8 ± 24.4 ng [mg protein]^–1^ h^–1^; *p* = 0.006; Fig. 6) compared with NT cells. This suggests that E16 infection perturbs beta cell function.

### E16 infection disrupts autophagic flux in human islet cells

**Fig. 7:**
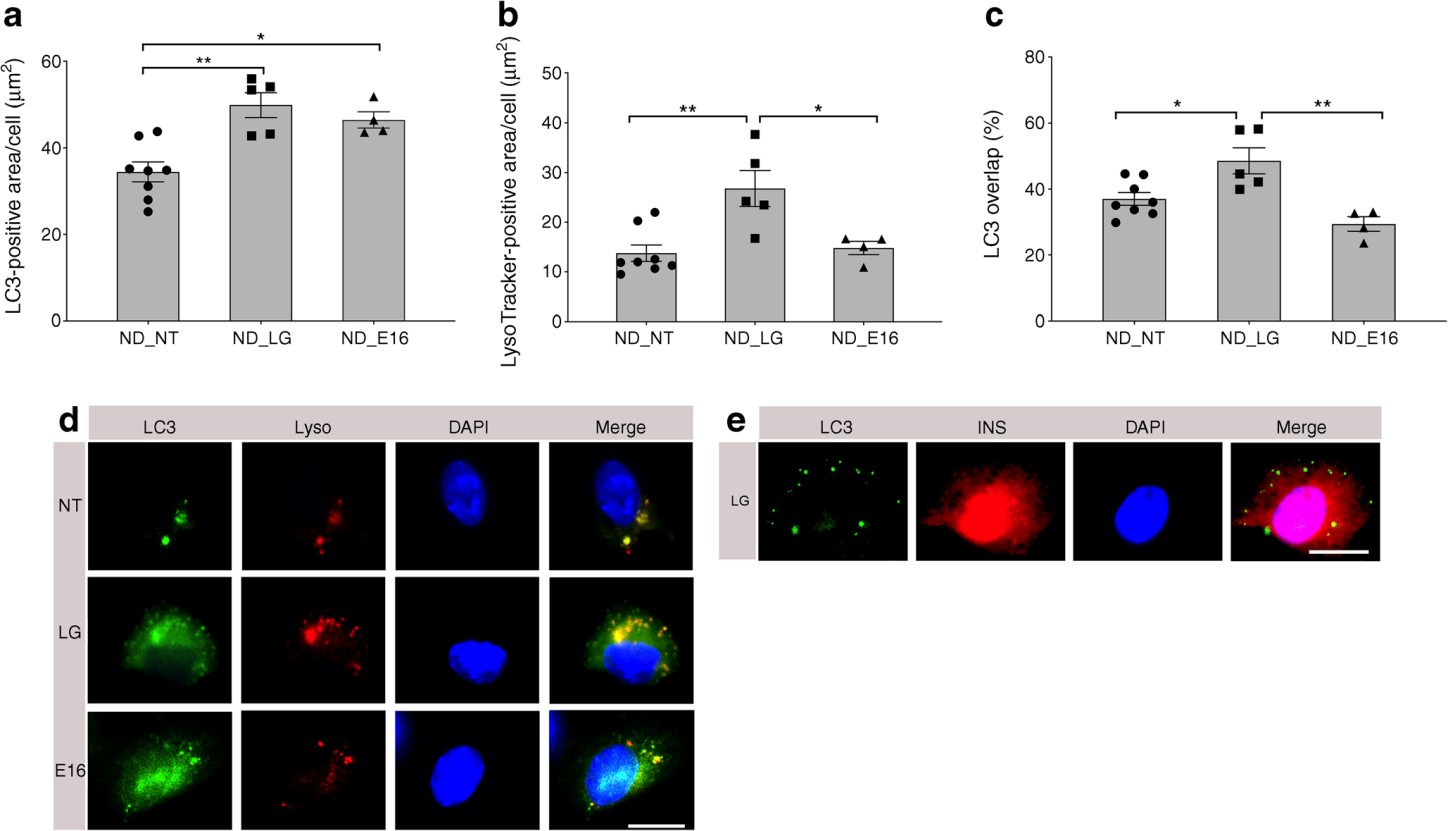
Autophagosome and lysosome detection in E16-infected human islet cells. Quantification of LC3II-GFP-positive area per cell (μm^2^) in non-diabetic (ND)_NT, ND_LG or ND_E16-infected cells (**a**). Quantification of LysoTracker-positive area per cell (μm^2^) in ND_NT, ND_LG or ND_E16-infected cells (**b**). Percentage of LC3II-GFP-positive areas overlapping LysoTracker-positive areas in ND_NT, ND_LG or ND_E16 infected cells (**c**). ND_NT, *n* = 8; ND_LG, *n* = 5; ND_E16, *n* = 4. Data are presented as mean ± SEM. *p < 0.05, **p < 0.01. Representative images showing immunofluorescence staining of LC3II, LysoTracker (Lyso) and DAPI (**d**) and LC3II, insulin and DAPI (**e**)

Similar to INS(832/13) cells, the number of LC3II-GFP-positive puncta (μm^2^/cell) increased when non-diabetic human islet cells were cultured in LG (49.9 ± 2.1 vs 34.4 ± 2.2; *p* = 0.001) or infected with E16 (46.5 ± 1.6 vs 34.4 ± 2.2; *p* = 0.01; Fig. 7a), compared with NT cells. LysoTracker-positive puncta (μm^2^/cell) were significantly increased in islet cells grown in LG compared with NT islet cells (26.8 ± 3.2 vs 13.8 ± 1.5; *p* = 0.003), whereas E16-infected cells, presented similar values to NT cells (Fig. 7b). While LG conditions led to a higher percentage of co-localisation of LC3II-GFP-positive puncta with LysoTracker (48 ± 3.5 vs 37.0 ± 1.8; *p* = 0.02), compared with NT islet cells, the number of LC3II-GFP-positive puncta overlapping with LysoTracker-positive areas in E16-infected islet cells was similar to that of NT cells. Representative images of LC3II-GFP and LysoTracker staining support the quantitative data (Fig. 7d). Co-staining with LC3II-GFP and insulin-positive islet beta cells is presented in Fig. 7e.

Collectively, this indicates that, unlike culturing human islet cells in LG, in which autophagosomes proceed to mature to autolysosomes, an E16 infection prevents autolysosome formation. This implies that E16 disrupts the autophagic process in human islet cells similar to that observed in INS(832/13) cells.

## Discussion

Herein, we demonstrate that enteroviral infection of INS(832/13) cells and human islet cells impairs autophagic flux resulting in intracellular accumulation of autophagosomes. This was further associated with an increased viral replication and reduced GSIS. As such, our data are highly relevant in the context of triggering events that may lead to type 1 diabetes onset. In fact, research supports the idea that enteroviral infections can establish persistent infections within beta cells [1–3], resulting in autoimmunity.

We selected the E16 strain to model the influence of an enteroviral infection on autophagy and beta cell function. Previous studies have shown that strains of E16, isolated from patients with meningitis, resulted in the development of diabetes-related islet autoantibodies [18, 19, 25]. In addition, E16 is able to replicate in explanted human islets and clonal beta cell lines, thus concluding that E16 can target pancreatic endocrine cells [26, 27].

Autophagy is a dynamic process where autophagosomes are continually formed and degraded. Accumulation of autophagosomes could result either from increased formation, decreased maturation and autophagosome turnover, or reduced fusion with lysosomes. Our results suggest that, rather than increasing autophagosome formation, E16 infection inhibits processing of the autophagosomes by lysosomes. Indeed, our study shows that autophagosomes accumulate in the cytoplasm of E16-infected cells to the same extent as when cells were treated with the lysosomal inhibitor chloroquine.

Enteroviral infections are capable of changing the expression of several host genes [28]. We only observed a significant increase of *Atg7*. *Atg7* reportedly is crucial in formation of autophagosomes and full body knockouts of *Atg7* are neonatally lethal [29]. This supports the notion that autophagy is modulated by post-translational modifications [30–32]*.* Since enteroviruses rely on protein synthesis of host cells to support replication, it is likely that many autophagy-related proteins are subjected to post-translational modifications during viral infection. Whether such post-translational modifications occur in human islets and beta cell lines infected with E16 is an area we are currently investigating. Despite the fact that enteroviral infection could deregulate multiple proteins involved in autophagosome fusion [10] we were unable to detect such changes, which may represent a limitation to the study.

Interestingly, studies have demonstrated that double-membraned vesicles derived from the autophagosomal pathway may serve as scaffolds for viral replication [30, 31]. This would explain why E16 hampers autophagy flux, as autophagosome degradation would lead to loss of membrane sources for the assembly of enterovirus RNA replication complexes. Inevitably, we detected dsRNA and newly produced extracellular viral particles in the culture supernatant of E16-infected cells 24 hpi, suggesting that accumulation of autophagosomes provides an advantage for viral replication. Regardless of viral replication, INS(832/13) cells remained viable and did not undergo apoptosis, demonstrating that E16 does not require cell lysis to egress INS(832/13) cells. Although enteroviruses are typically considered cytolytic viruses that kill host cells to release virus particles, it is likely that E16-bearing autophagosomes can bypass lysosomal degradation and are released from INS(832/13) cells in a non-lytic fashion [33–36]. The non-lytic release of infectious virus within secretory autophagic vesicles is termed ‘Autophagosome-mediated exit With Out Lysis’ (AWOL) [37–39]. This strategy may represent a Trojan horse, enabling spread of enteroviruses in persistently infected beta cells, with minimised exposure to the immune system. If true, this phenomenon could explain the presence of enteroviral antigens in 60–70% of islets of patients with recent-onset type 1 diabetes [40–43].

Dysfunction of autophagy following E16 infection of INS(832/13) cells not only resulted in an increased accumulation of autophagosomes but also the accumulation of selective autophagic cargo, such as p62, which target ubiquitinated proteins for autophagic degradation. It is well known that ubiquitination of proteins and accumulation of damaged organelles is toxic for pancreatic beta cells [44]. Thus, besides creating an environment advantageous for viral replication, it is likely that dysfunctional autophagy leads to impaired clearance of toxic protein aggregates, thus contributing to beta cell dysfunction and type 1 diabetes progression. In fact, one study shows that deregulated autophagy in beta cells results in impaired glucose-induced cytosolic calcium signalling and, consequently, reduced insulin secretion [45]. In line with this, we demonstrate that E16 infection in INS(832/13) cells results in reduced GSIS, without any overt cell death. Notably, increased detection of LC3II-positive puncta and enhanced mRNA expression of *Atg* genes in INS(832/13) cells treated with either LG or EBSS, reinforces the idea that beta cell survival is heavily dependent on ample glucose concentrations. It also provides validation for the use of glucose-starved cells as a positive control for autophagy in our study.

In conclusion, we have investigated autophagy in clonal INS(832/13) cells and human islet cells infected with a strain of enterovirus, associated with islet autoimmunity. Our data provide compelling evidence that enteroviruses subvert autophagy for proviral purposes by disrupting the later stages of the autophagic pathway. We suggest that inhibition of autophagic turnover, in this case, is a virus-driven process that promotes viral replication and a non-lytic release, but also hampers beta cell function.

Future studies are warranted to determine the molecular mechanisms by which enteroviruses hijack autophagic pathways permitting effective viral replication and foster beta cell dysfunction. This information will not only shed light on mechanisms of viral infections that trigger type 1 diabetes, per se, but may identify novel antiviral strategies to therapeutically modulate autophagy to treat the disease.

## Electronic supplementary material

ESM (PDF 394 kb)

## Data Availability

The datasets generated during and/or analysed during the current study are available from the corresponding author upon reasonable request.

## Abbreviations

ATG: Autophagy-related
a.u.: Arbitrary units
BCA: Bicinchoninic acid
CCID_50_: 50% cell culture infectious dose
dsRNA: Double-stranded RNA
E16: Echovirus 16
EBSS: Earle’s Balanced Salt Solution
GMK: Green monkey kidney
GSIS: Glucose-stimulated insulin secretion
hpi: Hours post infection
LAMP2: Lysosome-associated membrane protein 2
LC3: Microtubule-associated protein light chain 3
LDH: Lactate dehydrogenase
LG: Low glucose (2.8 mmol/l)
MOI: Multiplicity of infection
MTT: 3-(4,5-Dimethylthiazol-2-yl)-2,5-diphenyltetrazolium bromide
NT: Non-treated (cell)
qPCR: Quantitative RT-PCR
