# Supplementary material for: Human enteroviral infection impairs autophagy in clonal INS(832/13) cells and human pancreatic islet cells
Source: Diabetologia. 2020 Jul 16;63(11):2372–84. doi: 10.1007/s00125-020-05219-z (PMC7527364; doi:10.1007/s00125-020-05219-z)
Supplement: Supplementary file 1 — (PDF 394 kb) [file 125_2020_5219_MOESM1_ESM.pdf]

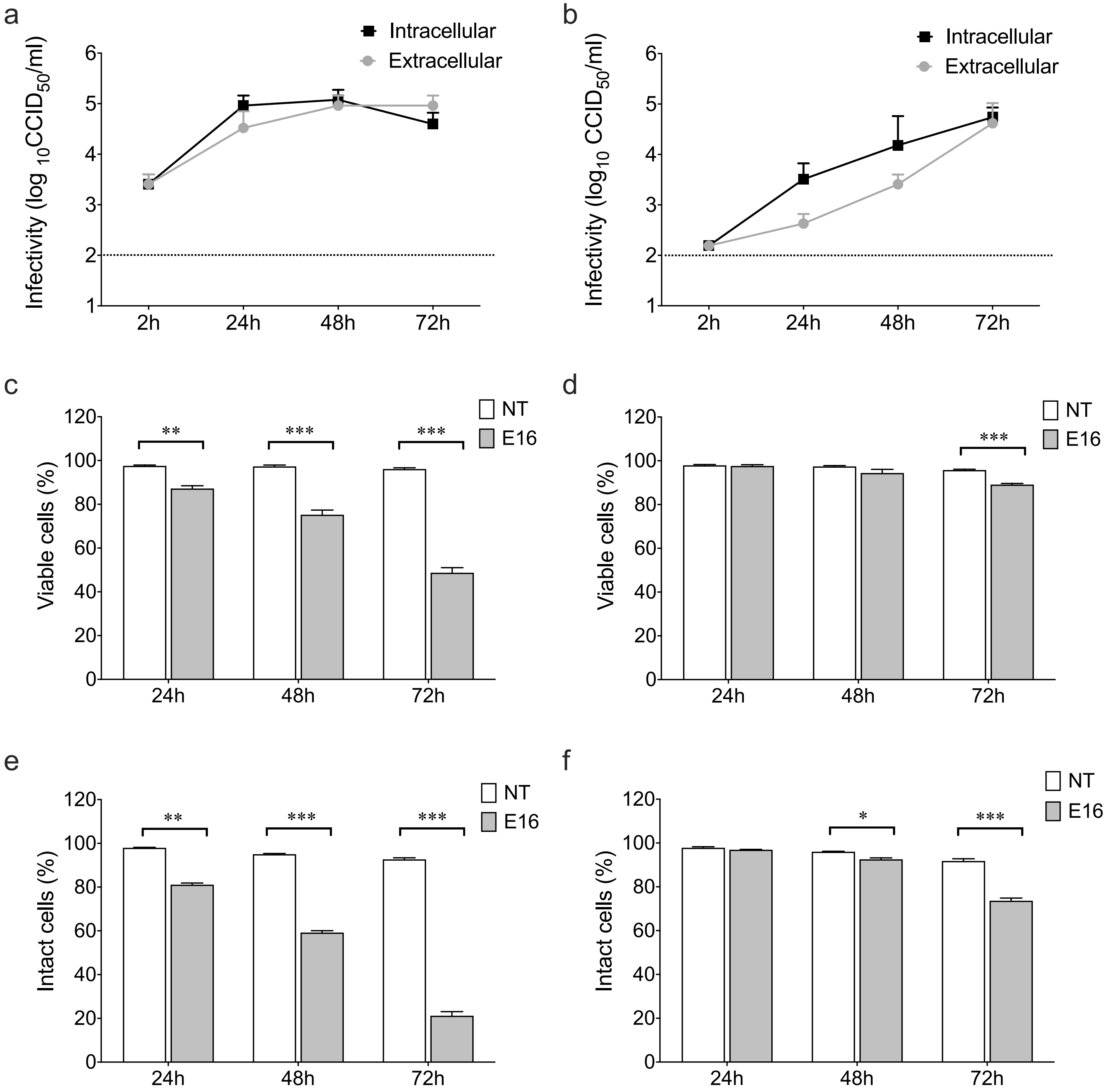

ESM Fig 1. Viral replication kinetics, viability and plasma membrane integrity analysis in E16 infected (MOI of 1 and 0.01) INS(832/13) cells MOI of 1 and 0.01. Intracellular virus production and extracellular virus release was determined by a dilution assay at different time points (2, 24, 48 and 72h) after infection with E16 at MOI 1 **(a)** and 0.01 **(b)**, dotted black lines indicate the limit of detection. Cell viability (MTT assay) at indicated time points after E16 infection at MOI 1 **(c)** and 0.01 **(d)** compared to NT cells. Plasma membrane integrity determined by LDH leakage at the indicated time points after E16 infection at MOI 1 **(e)** and 0.01 **(f)** compared to NT cells. Results include data from three independent experiments, with each measurement performed in triplicate. Data are presented as mean ± SEM * $p < 0.05$, ** $p < 0.01$, *** $p < 0.001$.

ESM Figure 1. Viral replication kinetics, viability and plasma membrane integrity analysis in E16 infected (MOI of 1 and 0.01) INS(832/13) cells MOI of 1 and 0.01. Intracellular virus production and extracellular virus release was determined by a dilution assay at different time points (2, 24, 48 and 72h) after infection with E16 at MOI 1 (**a**) and 0.01 (**b**), dotted black lines indicate the limit of detection. Cell viability (MTT assay) at indicated time points after E16 infection at MOI 1 (**c**) and 0.01 (**d**) compared to NT cells. Plasma membrane integrity determined by LDH leakage from cell culture supernatant at the indicated time points after E16 infection at MOI 1 (**e**) and 0.01 (**f**) compared to NT cells. Results include data from three independent experiments, with each measurement performed in triplicate. Data are presented as mean ± SEM * $p < 0.05$, *** $p < 0.001$.

Checklist for reporting human islet preparations used in research

Adapted from Hart NJ, Powers AC (2018) Progress, challenges, and suggestions for using human islets to understand islet biology and human diabetes. Diabetologia https://doi.org/10.1007/s00125-018-4772-2

**Islet preparation**	**1**	**2**	**3**	**4**	**5**	**6**	**7**	**8[a]**
**MANDATORY INFORMATION**								
Unique identifier	246	247	248	249	250	252	255	256
Donor age (years)	39	67	43	64	59	58	57	61
Donor sex (M/F)	M	M	M	F	F	F	M	M
Donor BMI (kg/m^2)	24,7	32,5	30,9	26,9	29,4	Not available	30,4	37
Donor HbA_{1c} or other measure of blood glucose control	5,8% 39.9mmol/mol	5,8% 39.9mmol/mol	5,8% 39.9mmol/mol	5,7% 38.8mmol/mol	6,6% 48.6mmol/mol	5,9% 41.0mmol/mol	Not available	6,1% 43.2mmol/mol
Origin/source of islets[b]	Scandinavia	Scandinavia	Scandinavia	Scandinavia	Scandinavia	Scandinavia	Scandinavia	Scandinavia
Islet isolation centre	Nordic Network for Clinical transplantation, Uppsala	Nordic Network for Clinical transplantation, Uppsala	Nordic Network for Clinical transplantation, Uppsala	Nordic Network for Clinical transplantation, Uppsala	Nordic Network for Clinical transplantation, Uppsala	Nordic Network for Clinical transplantation, Uppsala	Nordic Network for Clinical transplantation, Uppsala	Nordic Network for Clinical transplantation, Uppsala
Donor history of diabetes? Please select yes/no from drop down list	No	No	No	No	No	No	No	No
**If Yes, complete the next two lines if this information is available**								
Diabetes duration (years)								
Glucose-lowering therapy at time of death[c]								
**RECOMMENDED INFORMATION**								

Donor cause of death	Not available	Not available	Not available	Not available	Not available	Not available	Not available	Not available
Warm ischaemia time (h)	Not available	Not available	Not available	Not available	Not available	Not available	Not available	Not available
Cold ischaemia time (h)	Not available	Not available	Not available	Not available	Not available	Not available	Not available	Not available
Estimated purity (%)	84	60	94	49	21	45	40	85
Estimated viability (%)	Not available	Not available	Not available	Not available	Not available	Not available	Not available	Not available
Total culture time (h)[d]	4	3	2	5	5	4	4	4
Glucose-stimulated insulin secretion or other functional measurement[e]	32	9.3	14.2	2.9	13.2	9.8	2.4	2.8
Handpicked to purity? Please select yes/no from drop down list	Yes	Yes	Yes	Yes	Yes	Yes	Yes	Yes
Additional notes	e) Secretory index is calculated as fold increase between low (1mM) and high (20mM) glucose in a dynamic perifusion setup.							

[a]If you have used more than eight islet preparations, please complete additional forms as necessary
[b]For example, IIDP, ECIT, Alberta IsletCore
[c]Please specify the therapy/therapies
[d]Time of islet culture at the isolation centre, during shipment and at the receiving laboratory
[e]Please specify the test and the results

Checklist for reporting human islet preparations used in research

Adapted from Hart NJ, Powers AC (2018) Progress, challenges, and suggestions for using human islets to understand islet biology and human diabetes. Diabetologia https://doi.org/10.1007/s00125-018-4772-2

**Islet preparation**	**1**	**2**	**3**	**4**	**5**	**6**	**7**	**8[a]**
**MANDATORY INFORMATION**								
Unique identifier	303	304	306					
Donor age (years)	39	70	75					
Donor sex (M/F)	M	F	F					
Donor BMI (kg/m^2)	26,6	40,8	31,1					
Donor HbA_{1c} or other measure of blood glucose control	5.3% 34.4mmol/mol	6.0% 42.1mmol/mol	5.4% 35.5mmol/mol					
Origin/source of islets[b]	Scandinavia	Scandinavia	Scandinavia					
Islet isolation centre	Nordic Network for Clinical transplantation, Uppsala	Nordic Network for Clinical transplantation, Uppsala	Nordic Network for Clinical transplantation, Uppsala					
Donor history of diabetes? Please select yes/no from drop down list	No	No	No					
**If Yes, complete the next two lines if this information is available**								
Diabetes duration (years)								
Glucose-lowering therapy at time of death[c]								
**RECOMMENDED INFORMATION**								

Donor cause of death	Not available	Not available	Not available						
Warm ischaemia time (h)	Not available	Not available	Not available						
Cold ischaemia time (h)	Not available	Not available	Not available						
Estimated purity (%)	78	94	95						
Estimated viability (%)	Not available	Not available	Not available						
Total culture time (h)[d]	3	4	4						
Glucose-stimulated insulin secretion or other functional measurement[e]	18	1.6	2.3						
Handpicked to purity? Please select yes/no from drop down list	Yes	Yes	Yes						
Additional notes	e) Secretory index is calculated as fold increase between low (1mM) and high (20mM) glucose in a dynamic perifusion setup.								

[a]If you have used more than eight islet preparations, please complete additional forms as necessary
[b]For example, IIDP, ECIT, Alberta IsletCore
[c]Please specify the therapy/therapies
[d]Time of islet culture at the isolation centre, during shipment and at the receiving laboratory
[e]Please specify the test and the results
